# Response Surface Methodology Optimization of Electron-Beam-Irradiated Carboxymethyl Cellulose/Citric Acid-Based Hydrogels

**DOI:** 10.3390/gels11110928

**Published:** 2025-11-19

**Authors:** Sa Rang Choi, Jung Myoung Lee

**Affiliations:** Department of Wood and Paper Science, Kyungpook National University, 80 Daehakro, Daegu 41566, Republic of Korea; luvvchoi@knu.ac.kr

**Keywords:** electron beam irradiation, carboxymethyl cellulose, citric acid, hydrogel, response surface methodology, central composite design

## Abstract

Electron beam irradiation (EBI) is an environmentally friendly cross-linking technique that can form covalent bonds between natural polymers without the use of chemical cross-linkers. In this study, carboxymethyl cellulose (CMC; 3000 cPs) and citric acid (CA) were used to prepare hydrogels under low-dose EBI conditions (7 kGy). The effects of composition variables were statistically analyzed using response surface methodology based on central composite design. The concentrations of CMC (4–14 wt%) and CA (1–4 wt%) were selected as independent variables, while the gel fraction, water absorption, and elastic modulus were employed as responses. Analysis of variance confirmed that the quadratic models were statistically significant (*p* < 0.05) with a high predictive reliability (R^2^ = 0.91–0.98). Statistical validation demonstrated that the residuals were normally distributed and that all data fell within the 95% prediction interval, verifying the robustness of the model. Multi-response optimization identified an optimal composition of 8.88 wt% CMC and 0.03 wt% CA, yielding a predicted gel fraction of 88.7%, water absorption of 256 g/g, and modulus of 2273 Pa. The extended condition (CMC 9.12 wt%, CA 2.17 × 10^−7^ wt%) achieved similar absorbency with a ~9% higher modulus. This study established a reliable predictive model correlating the composition and properties of EBI-induced CMC–CA hydrogels, providing a foundation for optimizing eco-friendly hydrogel processes and scaling them up in the future.

## 1. Introduction

Highly absorbent hydrogels are functional materials with a three-dimensional (3D) network structure composed of hydrophilic polymers. They can absorb several hundred times their weight in water while maintaining their structural integrity [1,2,3]. These properties allow hydrogels to be widely utilized in various applications such as drug delivery systems, wound dressings, tissue engineering scaffolds, sanitary products, and agricultural water retention agents [4,5,6]. Most commercially available superabsorbent polymers (SAPs) are synthesized from poly(acrylic acid)-based polymers derived from petrochemical feedstocks, which are difficult to biodegrade. The potential environmental and human health risks associated with residual monomers and chemical cross-linkers have raised growing concerns [7,8].

To overcome these limitations, the development of eco-friendly hydrogels based on renewable, biomass-derived polymers has attracted considerable attention. Examples include cellulose, chitosan, alginate, starch, and natural polysaccharide gums. Of these, carboxymethyl cellulose (CMC) is considered one of the most promising bio-based materials due to its excellent hydrophilicity, biodegradability, biocompatibility, and low toxicity. CMC, in which the hydroxyl groups (–OH) of cellulose are substituted with carboxymethyl groups, readily dissolves in water, has high viscosity, and demonstrates strong chemical reactivity, enabling the formation of stable and flexible hydrogel networks through various cross-linking reactions [9,10,11].

The stability, mechanical strength, and swelling degree of hydrogels are all strongly affected by the crosslinking mechanisms involved. Crosslinking can be classified into physical crosslinking (e.g., hydrogen bonding and ionic interactions) and chemical crosslinking (e.g., covalent bonding) [12]. Although physical crosslinking offers excellent biocompatibility because it does not involve chemically toxic reagents, the resulting networks are often sensitive to external environmental factors such as pH and temperature. In contrast, chemical crosslinking forms stable 3D networks using agents such as epichlorohydrin, glutaraldehyde, or N,N′-methylenebisacrylamide (MBA), but these reagents raise safety concerns related to toxicity and residual contamination, limiting their use in biomedical and food applications [13,14].

As an alternative, catalyst-free crosslinking techniques based on radiation—such as electron beam (EB), gamma-ray, or ultraviolet (UV) irradiation—have attracted increasing attention. EB irradiation (EBI) induces the formation of interchain covalent bonds by generating free radicals within the polymer matrix. This approach eliminates additional chemical catalysts or purification steps, provides rapid reaction kinetics, and allows precise process control. Compared with gamma irradiation, EB offers shorter irradiation times, lower thermal effects, and greater suitability for process automation. When CMC is irradiated with EB, hydroxyl radicals, hydrated electrons, and hydrogen radicals are produced in the aqueous phase, which subsequently react with the hydroxyl and carboxyl groups of CMC to form covalent crosslinks. As a result, a stable network structure can be achieved without using toxic chemical crosslinkers [15,16,17].

Radiation-induced crosslinking has been applied to various natural polymer systems. For example, in a study by Shin (2015) [18], citric acid (CA), a natural organic acid derived from citrus fruits, was employed as an eco-friendly crosslinking agent. A CMC hydrogel with a high gel fraction (95%) and superior water absorption capacity (7000%) was successfully synthesized using EBI. The crosslinking mechanisms that occur between CMC and CA under EBI are illustrated in Figure S1. Crosslinking in the CMC/CA system mainly arises from esterification reactions between the hydroxyl groups of CMC and the carboxyl groups of CA, as well as from reactions between the functional groups of adjacent CMC chains. During thermal or EB treatment, partial dehydration occurs between the carboxyl and neighboring hydroxyl groups of CMC, forming covalent ester linkages and additional hydrogen-bonding networks that reinforce the gel structure [18,19]. However, excessive irradiation may cause chain scission, reducing the structural integrity of the gel network. It has been reported that relatively low irradiation doses (<10 kGy) can initiate crosslinking reactions rather than chain scission, whereas higher doses tend to promote chain cleavage due to the rupture of glycosidic linkages [20]. To date, most studies have been conducted under high irradiation doses (≥10 kGy) [21], while research on the crosslinking efficiency and variation in the material properties under low-dose conditions (<10 kGy) remains limited. Under low-dose EBI, it is likely that hydroxyl radicals and hydrated electrons generated by radiolysis will promote a similar dehydration-condensation reaction without catalysts or high temperatures, leading to efficient cross-linking while minimizing polymer degradation.

Therefore, it is essential to explore the optimal hydrogel composition under low-dose irradiation. This study used low-dose EBI to prepare hydrogels by irradiating mixtures of high-viscosity CMC (5000 cPs) and CA as a natural crosslinking agent [22]. The effects of CMC concentration and the presence or absence of CA on the properties of the resulting hydrogel were investigated. Crosslinked networks were not formed without CA, regardless of CMC concentration. In contrast, adding CA significantly enhanced the crosslinking efficiency with increasing CMC content, yielding hydrogels with a maximum gel fraction of 68% and a water absorption capacity of 170 g/g. These findings indicate that EB crosslinking can achieve sufficient reactivity even at low doses.

The intrinsic properties of CMC precursor (e.g., its viscosity, molecular weight, and degree of substitution) strongly influence the physical characteristics of the resulting hydrogel [23,24]. However, high-viscosity CMC has poor stirrability at elevated concentrations, limiting its processability. Based on previous findings, the present study statistically analyzed the effects of CMC and CA concentrations on gel formation and water absorption under low-dose (7 kGy) EBI using medium-viscosity CMC (3000 cPs), which exhibits approximately 40% lower viscosity than that used in prior experiments.

Central composite design (CCD) is a form of response surface methodology (RSM) that enables the analysis of correlations between experimental factors and response variables. This statistical approach can be used to efficiently determine, with a minimal number of experiments, how the interactions between two or more variables influence the response [25,26,27]. In this study, CCD was employed to optimize the mixing conditions for CMC and CA. The concentrations of CMC (4–14 wt%) and CA (1–4 wt%) were defined as independent variables. Based on the experimental results, a quadratic regression model was established to assess the model’s adequacy and to simultaneously identify the optimal combination of the gel fraction, water absorption, and elastic modulus.

This study provides a scientific basis for the development of sustainable superabsorbent materials by demonstrating an environmentally benign approach to fabricating high-performance hydrogels without chemical crosslinkers. The findings are expected to contribute to the broader application of eco-friendly hydrogels in medicine, agriculture, and sanitation.

## 2. Results and Discussion

### 2.1. ANOVA Results and Model Significance

In this study, the concentrations of CMC and CA were selected as independent factors, and response surface analysis was conducted using CCD. Based on the experimental results (Table S1), quadratic regression models were developed for each response variable (i.e., the gel fraction, water absorption, and modulus), which were then evaluated using analysis of variance (ANOVA). According to the model fit summaries (Tables S2–S4), the quadratic model provided the best statistical performance, producing higher R^2^ values than the linear or two-factor interaction (2FI) models. The cubic model was excluded to avoid overfitting. In particular, all of the quadratic models were significant (*p* < 0.05), indicating that they effectively explained the experimental variation [27].

For the gel fraction (Table 1), the overall model was significant (*p* = 0.0012), as were the main effects of CMC (*p* = 0.0004), CA (*p* = 0.0024), and the quadratic term CMC^2^ (*p* = 0.0062). However, the interaction (CMC × CA, *p* = 0.9798) and CA^2^ (*p* = 0.9264) were not significant, suggesting that gel formation mainly depended on the individual concentrations of CMC and CA.

For water absorption (Table 2), the overall model was highly significant (*p* < 0.0001). The CA concentration had the greatest influence (*p* < 0.0001), confirming its key role in swelling behavior. Although CMC alone was not significant (*p* = 0.4519), its interaction with CA (*p* = 0.0310) was significant, indicating that maximum absorption occurred at specific CMC/CA ratios. The quadratic term CMC^2^ (*p* < 0.0001) revealed a nonlinear trend, with peak absorption near an optimal CMC concentration [28].

The modulus model also exhibited strong significance (F = 67.27, *p* < 0.0001; Table 3). Both CMC (*p* < 0.0001) and CA (*p* = 0.0241) had significant main effects, while their interaction (*p* = 0.0298) was also significant, indicating that the modulus is jointly influenced by both factors. The quadratic term for CMC^2^ was highly significant (*p* = 0.0008), reflecting a nonlinear dependence on CMC concentration, whereas CA^2^ was not (*p* = 0.5602). These results suggest that CMC primarily determines network stiffness, which becomes saturated at high concentrations, while CA acts as an effective crosslinking agent at moderate levels.

### 2.2. Statistical Evaluation of Model Fit

Table 4 summarizes the fit statistics of the quadratic models for the gel fraction, water absorption rate, and modulus. The R^2^ ranged from 0.91 to 0.98 for all responses, confirming the statistical validity of the quadratic models [29]. Notably, the differences between adjusted R^2^ and predicted R^2^ were below 0.20, indicating excellent predictive reliability [30]. The adequate precision values also exceeded the recommended threshold (>4) for all responses (12.46–25.28), demonstrating a sufficient signal-to-noise ratio [31]. Although the lack-of-fit *p*-values were below 0.05, the deviation was not statistically significant compared with the pure error, suggesting that the quadratic models adequately described the nonlinear behavior of each response variable.

**Figure 1:**
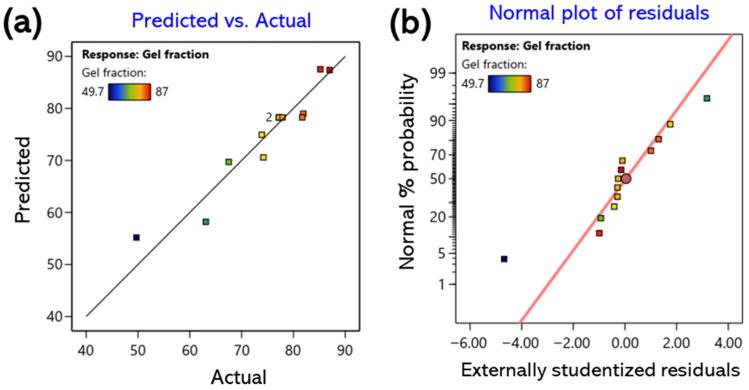
Response surface for the gel fraction showing the interactive effects of CMC and CA concentrations; (**a**) predicted vs. actual values for model validation; (**b**) normal plot of residuals.

**Figure 2:**
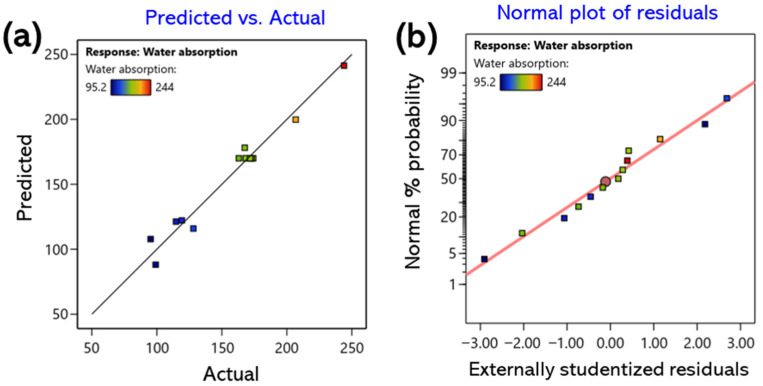
Response surface for water absorption showing the interactive effects of CMC and CA concentrations; (**a**) predicted vs. actual values for model validation; (**b**) normal plot of residuals.

**Figure 3:**
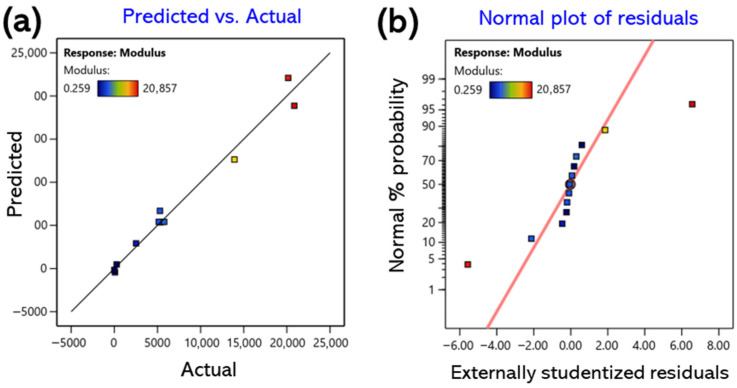
Response surface for the modulus showing the interactive effects of CMC and CA concentrations; (**a**) predicted vs. actual values for model validation; (**b**) normal plot of residuals.

The correlations between the predicted and experimental values are summarized in Figure 1a, Figure 2a and Figure 3a. Most data points were distributed near the 45° line, indicating a high level of agreement between the predicted and observed values. Furthermore, the standard probability plots of residuals (Figure 1b, Figure 2b and Figure 3b) exhibited a uniform distribution around the baseline, satisfying the normality assumption. These findings confirm the absence of systematic bias or outliers in the model and demonstrate that the developed regression model is statistically robust and reliable for predicting all response variables [32].

### 2.3. Regression Model and Coefficients

To quantitatively evaluate the influence of CMC and CA concentrations on the structural and physical properties of the hydrogels, regression analysis was conducted using a fitted quadratic model. This analysis quantifies the contribution of the main effect, interaction term, and squared term to the three key response variables (gel fraction, water absorption, and modulus) that govern the hydrogel’s structural and physical characteristics. The regression coefficients and corresponding *p*-values are summarized in Table 5, while the detailed coded values and actual regression coefficients used for model construction are provided in the Supplementary Materials (Tables S6 and S7).

For the gel fraction, the regression coefficient for CMC (+8.42) indicates that increasing CMC content enhances gelation due to denser interchain crosslinking at higher polymer concentrations [33]. In contrast, the negative coefficient for CA (−6.25) suggests that excess crosslinker reduces gelation, likely by lowering functional group reactivity or disrupting uniform network formation [34,35]. The quadratic term CMC^2^ (−5.59) also exhibited a nonlinear decrease, suggesting that polymer chain aggregation at high CMC concentrations limits crosslinking and induces nonuniform structures.

For water absorption, the CA concentration had a strong negative effect (−42.14), indicating reduced crosslinking efficiency when CA exceeds the optimal range. This aligns with the trend for the gel fraction, where excess CA saturates CMC reactive sites and leads to irregular micro-networks with more soluble fractions, decreasing the swelling capacity. Conversely, CMC showed a moderate positive effect (+2.87), reflecting its role in increasing the number of hydrophilic sites up to a critical level. However, the quadratic term (−29.08) confirmed a nonlinear decrease beyond this point due to chain aggregation. The positive interaction term (CMC × CA, +13.70) suggests a synergistic effect in which an optimal crosslinker concentration promotes uniform bonding and stable diffusion pathways within the hydrogel network.

For the modulus, CMC concentration (+7866.91) had the most dominant positive effect, confirming its major role in reinforcing the hydrogel structure through higher crosslink density and chain entanglement. The CA term (+1330.33) and CMC × CA interaction (+1783.93) also contributed positively, suggesting that moderate crosslinking improves network cohesion and elastic recovery. In contrast, the quadratic term CMC^2^ (+2780.40) indicates nonlinear stiffening due to internal stress accumulation at high CMC levels, whereas CA^2^ (−304.17) reflects brittleness caused by excessive crosslinker addition.

Regression equations (Equations (1)–(3)) were derived based on these findings to enable quantitative comparison of the relative influence of each factor:

(1) $$Gelfraction=78.26+8.42\times A+(-6.25)\times B+(-0.05)\times AB+(-5.59)\times A^{2}+0.14\times B^{2}$$

(2) $$Waterabsorption=170.02+2.87\times A+(-42.14)\times B+13.70\times AB+(-29.08)\times A^{2}+5.90\times B^{2}$$

(3) $$Modulus=5408.22+7866.91\times A+1330.33\times B+1783.93\times AB+2780.40\times A^{2}+(-304.17)\times B^{2}$$

where *A* and *B* represent the coded values of the independent variables corresponding to CMC and CA, respectively. The regression equations derived from the experimental values are presented in the Supplementary Materials (Equations (S1)–(S3)).

### 2.4. Response Surface Analysis

**Figure 4:**
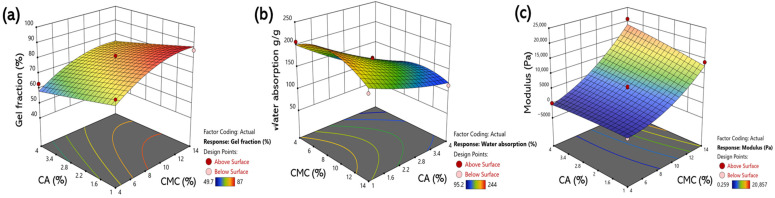
Three-dimensional response surface and contour plots for the (**a**) gel fraction; (**b**) water absorption; (**c**) modulus.

Figure 4 presents the 3D response surface and contour plots illustrating the interactive effects of CMC and CA concentrations on the gel fraction (Figure 4a), water absorption rate (Figure 4b), and modulus (Figure 4c). These plots clearly visualize the nonlinear variation resulting from the interaction between CMC and CA. Each model was predicted using the quadratic regression equations (Equations (1)–(3)) derived from the CCD and interpreted based on the previously validated statistical significance (Table 1, Table 2 and Table 3) and model fit statistics (Table 4).

For the gel fraction, values increased with both CMC and CA concentrations, with a stronger effect at higher CMC levels. CA enhanced crosslinking up to ≈ 2–3 wt%, but further increases caused a slight decline due to reduced crosslinking efficiency and network irregularity, which was consistent with the negative regression coefficient (−6.25) for CA. A typical nonlinear trend was observed for the water absorption. Swelling initially increased with CMC concentration but decreased beyond the optimum point. At low CMC levels, abundant hydrophilic groups enhanced swelling; however, excessive CMC or CA generated an inhomogeneous network that reduced the crosslinking efficiency and restricted water diffusion. Maximum absorption occurred at a specific CMC/CA ratio where the polymer and crosslinker content were balanced. For the modulus, CMC concentration had the dominant influence, with higher concentrations enhancing the crosslinking density and chain entanglement, strengthening the hydrogel network. CA also contributed positively through ester-type crosslinking, particularly in CMC-rich compositions. Overall, both CMC and CA exerted distinct nonlinear effects on all three responses. Within an optimal composition range, a high gel fraction, balanced swelling, and improved mechanical strength could thus be simultaneously achieved.

### 2.5. Model Validation

To validate the predictive accuracy of the quadratic regression model, hydrogels were prepared under two conditions: (i) CMC 9 wt%/CA 0.38 wt% and (ii) CMC 9 wt%/CA 4 wt%. Their properties were experimentally measured and compared with model predictions (Table 6; Tables S10 and S11).

Under CMC 9/CA 0.38 condition, the predicted gel fraction, water absorption, and modulus were 87.4%, 241 g/g, and 2920 Pa, respectively, while the measured values were 87.5%, 211 g/g, and 2967 Pa. For CMC 9/CA 4, the gel fraction increased slightly, but water absorption decreased by ≈18%, and the modulus was 4.6% lower (6434 Pa predicted vs. 6137 Pa measured). This variation was the result of the high CA content, which reduced the crosslinking efficiency and produced heterogeneous networks containing both over- and under-crosslinked regions. This heterogeneity limited swelling and reduced water uptake, while the higher solid content (≈13%) created a denser structure that improved stiffness. Thus, increasing CA reinforced the network physically but hindered the chemical crosslinking efficiency. CMC-to-CA ratio is thus a key parameter controlling the trade-off between swelling and strength, requiring careful compositional balance to achieve optimal performance. There was close agreement between the predicted and measured values for the gel fraction and modulus (within 5%), while the water-absorption error (≈12–17%) remained within the 95% prediction interval [36]. These results confirm that the developed model reliably predicted the structural and mechanical behavior of CMC/CA hydrogel system.

### 2.6. Multi-Response Optimization

**Figure 5:**
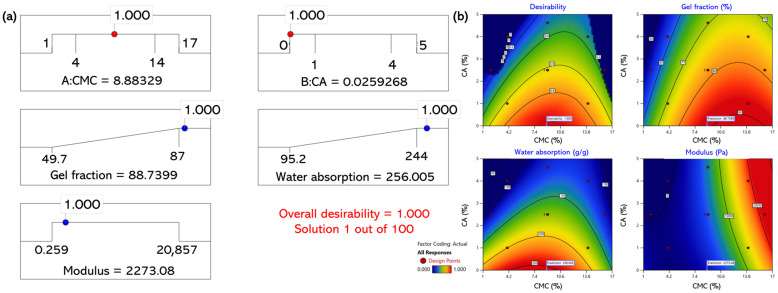
Multi-response optimization of CMC–CA hydrogel within the experimental range; (**a**) desirability-function optimization result; (**b**) response surface plots.

It is difficult to optimize one of the gel fraction, water absorption, or modulus without negatively affecting the others. Therefore, multi-response optimization was performed using the desirability function in Design-Expert. Detailed statistical parameters, including the 95% confidence interval (CI) and 99% tolerance interval (TI), are summarized in Tables S11 and S14). The gel fraction and water absorption were maximized, while the modulus was constrained within the experimental range. Figure 5a presents the optimization results within the experimental domain. The total desirability reached 1.000, indicating an excellent fit between the model and target criteria. The optimal composition was 8.88 wt% CMC and 0.03 wt% CA, with predicted values of 88.7% for the gel fraction, 256 g/g for the water absorption, and 2273 Pa for the modulus. As shown in Figure 5b, both the gel fraction and water absorption peaked when the CA concentration was ≤1 wt%. Increasing CMC enhanced the crosslinking and strength, whereas excessive CA reduced the efficiency.

**Figure 6:**
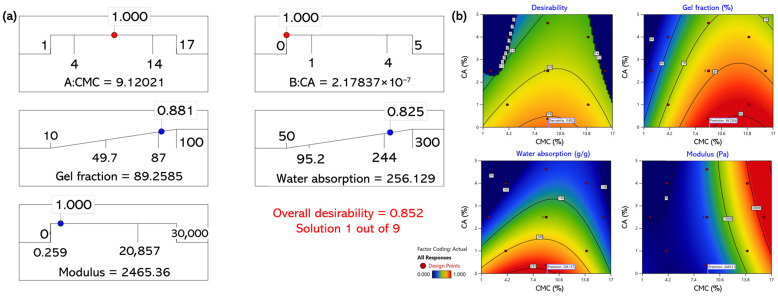
Multi-response optimization of CMC–CA hydrogel with extended target ranges; (**a**) desirability-function optimization result; (**b**) response surface plots.

Figure 6 shows the extended optimization beyond the initial design range. The model remained stable (overall desirability = 0.852), with an optimum at 9.12 wt% CMC and 2.17 × 10^−7^ wt% CA, giving predicted values of 89.2%, 256 g/g, and 2465 Pa for the gel fraction, water absorption, and modulus, respectively. This extremely low CA value was a theoretical and not experimentally realizable hydrogel formulation. Rather, it represented a mathematical prediction generated by the regression model to illustrate how the gel behavior changed as the CA content approached zero. The regression model developed in this study was not intended to specifically determine a single optimum but rather to predict compositional trade-offs between the gel fraction, water absorption, and viscoelastic modulus. Because these responses are interdependent (e.g., denser networks improve strength but reduce swelling). Accordingly, the optimal formulation may vary depending on the intended application.

Although this work focused on compositional effects under a fixed EB dose of 7 kGy, variation in dose uniformity may arise depending on the sample thickness, moisture content, and viscosity during irradiation. Hence, calibration is essential when scaling up the process. Future studies should examine the radical generation efficiency and crosslinking behavior across a broader dose range (7–30 kGy) and assess the feasibility of large-area manufacturing via a continuous roll-to-roll EB process. Collectively, this study provides foundational data supporting the environmentally benign scalability and industrial feasibility of EB-based hydrogel fabrication technology.

## 3. Conclusions

In the present study, CMC–CA hydrogels were fabricated via EB crosslinking. The effects of CMC and CA concentrations on the gel fraction, water absorption, and elastic modulus were statistically analyzed using RSM based on CCD. The quadratic regression models were statistically significant for all response variables (R^2^ = 0.91–0.98). Model validation confirmed that the residuals satisfied normality and that all data points were contained within the 95% prediction interval, verifying the model’s reliability.

Multi-response optimization within the experimental range identified an optimal composition of 8.88 wt% CMC and 0.03 wt% CA, which provided a balanced formulation achieving a gel fraction of 88.7%, water absorption of 256 g/g, and a modulus of 2273 Pa. To verify the model’s applicability, a composition beyond the experimental range (9.12 wt% CMC and 2.17 × 10^−7^ wt% CA) was also evaluated. Under this condition, the gel fraction and water absorption remained nearly constant, while the modulus increased by approximately 9%.

This study quantitatively elucidated the influence and statistical correlation of composition variables during EB-induced hydrogel formation, thereby establishing a predictive model for the design of eco-friendly, highly absorbent materials. Future work will analyze radical generation and crosslinking efficiency under varying irradiation doses (7–30 kGy) and assess the feasibility of continuous roll-to-roll EB processing.

## 4. Materials and Methods

### 4.1. Materials

CMC was purchased from Samchun Chemicals Co., Ltd. (Seoul, Republic of Korea). The CMC had a degree of substitution (DS) of 0.80–0.90 and a viscosity of 3000 cP in a 1 wt% aqueous solution at 25 °C. Preliminary tests showed that low-viscosity CMC (<1000 cP) resulted in poor gel integrity after EBI. Medium-viscosity CMC (3000 cP) was thus selected because it offered a balanced combination of solubility, processability, and chain entanglement suitable for stable hydrogel formation. CA (≥99.0%, chemical pure grade) was obtained from Daejung Chemicals & Metals Co., Ltd. (Siheung, Republic of Korea) and used without further purification.

### 4.2. Experimental Design and Statistical Analysis

Two-factor, five-level CCD was employed to determine the optimal preparation conditions for CMC/CA hydrogel based on the mixing ratio of CMC and CA. The independent variables were the concentrations of CMC (4–14 wt%) and CA (1–4 wt%), while the response variables included the gel fraction (%), water absorption (g/g), and viscoelastic modulus (Pa). The CCD consisted of 13 experimental runs comprising four factorial points, four axial points (α = 1.414), and five replicated center points (Table 7). The replicated center runs were used to estimate pure error and ensure the statistical reliability and reproducibility of the experimental data. All runs were randomized to minimize potential order and carry-over effects. The concentration range for CA (1–4 wt%) was selected based on preliminary gelation trials and previously reported [22] formulations that produced stable crosslinking behavior in CMC/CA systems.

A second-order polynomial regression model was employed to predict the response values based on the experimental data obtained from the CCD. In this study, the regression analysis was performed using a quadratic model, and its general form can be expressed as shown in Equation (4):

(4) $$Y=\;\beta_{0}+\sum_{i=1}^{k}\beta_{i}X_{i}+\;\sum_{i=1}^{k}\beta_{ii}{X^{2}}_{i}+\;\sum_{i<j}^{k}\beta_{ij}X_{i}X_{j}$$

where *X_i_* and *X_j_* denote the coded values of the independent variables, and *k* represents the number of independent variables. *Y* indicates the predicted response corresponding to the physical properties of the hydrogel, namely the gel fraction, water absorption, and modulus. The coefficients *β*_0_, *β_i_*, *β_ij_*, and *β_ii_* represent the regression coefficients for the intercept, linear, interaction, and quadratic terms, respectively.

The significance of the independent variables and the statistical goodness-of-fit of the regression model were evaluated using ANOVA. Models with *p*-values less than 0.05 were considered statistically significant, while those with an *R*^2^ greater than 0.8 were regarded as having good predictive accuracy. All experimental designs, regression analyses, and response surface visualizations were conducted using Design-Expert version 13 software (Stat-Ease Inc., Minneapolis, MN, USA).

### 4.3. Preparation of the Hydrogels

CMC and CA were dissolved in distilled water at concentrations determined by the CCD. To minimize bubble formation during mixing, the solutions were stirred at 300 rpm for 30 min under vacuum conditions using a vacuum desiccator (SW-VDS3000, Samwoo Engineering Co., Ltd., Gyeonggi, Republic of Korea) equipped with a magnetic stirrer (MS3030D, Mtops Scientific Equipment Co., Ltd., Tehran, Iran). The resulting CMC–CA mixture was poured into a Petri dish (diameter: 9 cm; thickness: 1 cm) and subsequently irradiated with an EB at a dose of 7 kGy for 7.32 s using an EB accelerator (MB10-20, GeV Co., Ltd., Chungbuk, Republic of Korea).

### 4.4. Analysis of the Hydrogel Properties

#### 4.4.1. Gel Content

To determine the gel content of the hydrogel, approximately 1 g of the sample was immersed in distilled water and stirred at 100 rpm for 24 h in a shaking incubator (SI-30, Labhouse, Daejeon, Republic of Korea) maintained at 25 °C. After swelling, the soluble fraction of the hydrogel was removed by filtration using a glass microfiber filter (Whatman, Grade GF/A, 47 mm diameter, Cytiva, Marlborough, MA, USA). The insoluble residue remaining on the filter was then dried at 105 °C for 24 h. The dried hydrogel was weighed, and the gel fraction was calculated according to Equation (5):

(5) $$Gel\;fraction\;\left(\%\right)=\;\;(W_{1}\;/\;W_{0})\;\times \;100\;$$

where *W*_0_ and *W*_1_ are the dry weights of the hydrogel before and after extraction, respectively.

#### 4.4.2. Water Absorption

Approximately 1 g of the prepared hydrogel was placed in a food-grade teabag (8 × 10 cm; TangShan QiangDa Packaging Co., Ltd., Tangshan, China) and immersed in distilled water at 25 °C for 24 h. After immersion, the surface water was gently removed, and the weight of the swollen hydrogel was measured. The water absorption capacity of the hydrogel, defined as the amount of water absorbed per unit dry weight, was calculated using Equation (6):

(6) $$Water\;absorption\;\left(g/g\right)=(W_{S}-W_{0})/W_{0}$$

where *W_s_* and *W*_0_ denote the weights of the swollen hydrogel after 24 h and the dried hydrogel before immersion, respectively.

#### 4.4.3. Viscoelastic Modulus

Hydrogel samples were cut into disks (diameter: 25 mm; thickness: 1 mm) and analyzed using a rotational rheometer (MCR 102e, Anton Paar, Graz, Austria) under amplitude-sweep conditions ranging from 0 to 10,000% strain at a frequency of 1 rad/s. The maximum storage modulus (G′) within the linear viscoelastic region was the representative modulus value. During the measurement, parallel-plate geometry (diameter: 25 mm) was employed, and the gap between the plates was fixed at 1 mm.

## Figures and Tables

**Table 1:** ANOVA results derived from the quadratic model for the gel fraction.

Source	Sum of Squares	df	Mean Square	F-Value	*p*-Value	Note
Model	1101.43	5	220.29	15.09	0.0012	significant
CMC	566.80	1	566.80	38.83	0.0004	
CA	312.21	1	312.21	21.39	0.0024	
CMC × CA	0.0100	1	0.0100	0.0007	0.9798	
CMC^2^	217.09	1	217.09	14.87	0.0062	
CA^2^	0.1339	1	0.1339	0.0092	0.9264	
Residual	102.18	7	14.60			
Lack of Fit	87.05	3	29.02	7.67	0.0390	significant
Pure Error	15.13	4	3.78			
Cor Total	1203.61	12				

**Table 2:** ANOVA results derived from the quadratic model for water absorption.

Source	Sum of Squares	df	Mean Square	F-Value	*p*-Value	Note
Model	21,567.13	5	4313.43	41.64	<0.0001	significant
CMC	65.71	1	65.71	0.6344	0.4519	
CA	14,204.03	1	14,204.03	137.13	<0.0001	
CMC × CA	750.76	1	750.76	7.25	0.031	
CMC^2^	5882.25	1	5882.25	56.79	0.0001	
CA^2^	241.85	1	241.85	2.33	0.1703	
Residual	725.06	7	103.58			
Lack of Fit	646.67	3	215.56	11	0.0211	significant
Pure Error	78.39	4	19.6			
Cor Total	22,292.19	12				

**Table 3:** ANOVA results derived from the quadratic model for the modulus.

Source	Sum of Squares	df	Mean Square	F-Value	*p*-Value	Note
Model	5.789 × 10^8^	5	1.158 × 10^8^	67.27	<0.0001	significant
CMC	4.951 × 10^8^	1	4.951 × 10^8^	287.65	<0.0001	
CA	1.416 × 10^7^	1	1.416 × 10^7^	8.23	0.0241	
CMC × CA	1.273 × 10^7^	1	1.273 × 10^7^	7.4	0.0298	
CMC^2^	5.378 × 10^7^	1	5.378 × 10^7^	31.24	0.0008	
CA^2^	6.436 × 10^5^	1	6.436 × 10^5^	0.3739	0.5602	
Residual	1.205 × 10^7^	7	1.721 × 10^6^			
Lack of Fit	1.182 × 10^7^	3	3.940 × 10^6^	68.96	0.0007	significant
Pure Error	2.285 × 10^5^	4	57137.21			
Cor Total	5.910 × 10^8^	12				

**Table 4:** Fit statistics of the quadratic models for each response.

Factor	Gel Fraction	Water Absorption	Modulus
Std. Dev.	3.82	10.18	1311.95
Mean	74.91	155.75	6932.05
C.V. %	5.1	6.53	18.93
R^2^	0.91	0.97	0.98
Adjusted R^2^	0.85	0.94	0.97
Predicted R^2^	0.47	0.79	0.86
Adequate Precision	12.46	22.17	25.28
Lack of Fit (*p*-values)	0.039	0.021	0.001

**Table 5:** Summary of regression analysis and significance of model terms for each response.

Factor	Intercept	CMC	CA	CMC × CA	CMC^2^	CA^2^
Gel Fraction	78.26	8.42	−6.25	−0.05	−5.59	0.14
*p*-value		0.0004	0.0024	0.9798	0.0062	0.9264
Water Absorption	170.02	2.87	−42.14	13.7	−29.08	5.9
*p*-value		0.4519	<0.0001	0.031	0.0001	0.1703
Modulus	5408.22	7866.91	1330.33	1783.93	2780.4	−304.17
*p*-value		<0.0001	0.0241	0.0298	0.0008	0.5602

**Table 6:** Comparison between predicted and actual values for the hydrogel responses at selected conditions.

Factor	Predicted Value			Actual Value		
	Gel Fraction	Water Absorption	Modulus	Gel Fraction	Water Absorption	Modulus
CMC 9/CA 0.38	87.4	241.4	2920.4	87.5	210.9	2967.2
CMC 9/CA 4	72.2	133.8	6434.4	74.7	110	6137.1

**Table 7:** CCD matrix presenting the coded and actual values of the independent variables used for hydrogel preparation.

Run	Coded Values (CMC, CA)	CMC (wt%)	CA (wt%)
1	(0, 0)	9.00	2.50
2	(0, +α ^1^)	9.00	4.62
3	(+1, +1)	14.00	4.00
4	(+α, −1)	14.00	1.00
5	(+α, 0)	16.07	2.50
6	(0, 0)	9.00	2.50
7	(0, −α)	9.00	0.38
8	(0, 0)	9.00	2.50
9	(−1, +1)	4.00	4.00
10	(0, 0)	9.00	2.50
11	(0, 0)	9.00	2.50
12	(−α, 0)	1.93	2.50
13	(0, 0)	9.00	2.50

^1^ α = 1.414 (for two-factor CCD).

## Data Availability

The original contributions presented in this study are included in the article. Further inquiries can be directed to the corresponding author.

## Supplementary Materials

The following supporting information can be downloaded at: https://www.mdpi.com/article/10.3390/gels11110928/s1, Figure S1: Cross-liking mechanism between CMC and CA under electron-beam irradiation; Table S1: Experimental matrix and observed responses; Table S2: Fit Summary of gel fraction; Table S3: Fit Summary of response water absorption; Table S4: Fit Summary of modulus; Table S5: Model comparison statistics for gel fraction, water absorption, and modulus; Table S6: Coded regression coefficients of the quadratic models for gel fraction, water absorption, and modulus; Table S7: Actual regression coefficients of the quadratic models for gel fraction, water absorption, and modulus; Table S8: Regression coefficients and confidence intervals of the quadratic model for gel fraction (coded factors); Table S9: Regression coefficients and confidence intervals of the quadratic model for water absorption (coded factors); Table S10: Regression coefficients and confidence intervals of the quadratic model for modulus (coded factors); Table S11: Confirmation experiment results at the optimal condition (CMC 9 wt% / CA 0.38 wt%); Table S12: Confirmation experiment results at the optimal condition (CMC 9 wt% / CA 4 wt%); Table S13: Point prediction results and confidence intervals for the optimized CMC–CA hydrogel responses (Optimization results within experimental range); Table S14: Point prediction results and confidence intervals for the optimized CMC–CA hydrogel responses (Extended-range optimization results).

## Abbreviations

The following abbreviations are used in this manuscript:

ANOVA	Analysis of Variance
CA	Citric Acid
CCD	Central Composite Design
CI	Confidence Interval
CMC	Carboxymethyl Cellulose
DF	Degree of Freedom
DS	Degree of Substitution
EBI	Electron Beam Irradiation
EB	Electron Beam
MBA	N,N′-Methylenebisacrylamide
PI	Prediction Interval
RSM	Response Surface Methodology
TI	Tolerance Interval
UV	Ultraviolet
2FI	Two-Factor Interaction
3D	Three-dimensional
