# Supplementary material for: Response Surface Methodology Optimization of Electron-Beam-Irradiated Carboxymethyl Cellulose/Citric Acid-Based Hydrogels
Source: Gels. 2025 Nov 19;11(11):928. doi: 10.3390/gels11110928 (PMC12652089; doi:10.3390/gels11110928)
Supplement: Supplementary file 1 [file gels-11-00928-s001.zip › gels-3976222-supplementary.pdf]

Supplementary Materials

Response Surface Methodology Optimization of Electron-Beam Irradiated Carboxymethyl Cellulose/Citric Acid-Based Hydrogels

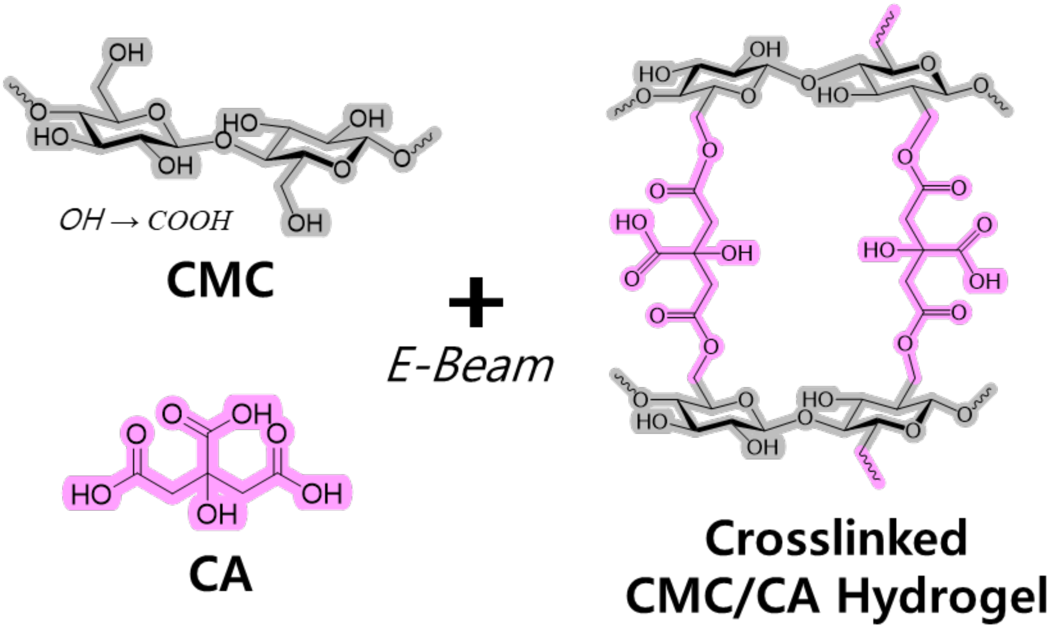

Figure S1. Cross-liking mechanism between CMC and CA under electron-beam irradiation.

Table S1. Experimental matrix and observed responses

Run	Factor		Response		
	CMC (wt%)	CA (wt%)	Gel fraction (%)	Water absorption (g/g)	Modulus (Pa)
1	9.00	2.50	77.2	168.3	5489
2	9.00	4.62	67.5	119.2	5286.6
3	14.00	4.00	73.9	114.7	20857
4	14.00	1.00	85.2	167.6	13921
5	16.07	2.50	81.9	128.1	20149
6	9.00	2.50	81.7	171.8	5779
7	9.00	0.38	74.2	206.8	268.52
8	9.00	2.50	77.2	174.1	5333.2
9	4.00	4.00	63.1	99.1	68.82
10	9.00	2.50	77.9	172.8	5156.6
11	9.00	2.50	87	244	2524.4
12	1.93	2.50	49.7	95.2	0.259
13	9.00	2.50	77.3	163.1	5283.3

Table S2. Fit Summary of gel fraction

Source	Sequential p-value	Lack of Fit p-value	Adjusted R^2	Predicted R^2	
Linear	0.0014	0.0122	0.6764	0.4660	
2FI	0.9871	0.0091	0.6404	0.3551	
Quadratic	0.0175	0.0390	0.8545	0.4661	Suggested
Cubic	0.0407	0.1345	0.9434	0.2752	Aliased

Table S3. Fit Summary of response water absorption

Source	Sequential p-value	Lack of Fit p-value	Adjusted R^2	Predicted R^2	
Linear	0.0060	0.0006	0.5681	0.2565	
2FI	0.3603	0.0005	0.5651	0.2359	
Quadratic	0.0003	0.0211	0.9442	0.7882	Suggested
Cubic	0.0039	0.8986	0.9915	0.9935	Aliased

Table S4. Fit Summary of modulus

Source	Sequential p-value	Lack of Fit p-value	Adjusted R^2	Predicted R^2	
Linear	< 0.0001	< 0.0001	0.8341	0.7155	
2FI	0.2296	< 0.0001	0.8444	0.6696	
Quadratic	0.0022	0.0007	0.9650	0.8572	Suggested
Cubic	0.2244	0.0005	0.9731	0.3064	Aliased

Table S5. Model comparison statistics for gel fraction, water absorption, and modulus

Factor	Gel fraction	Water absorption	Modulus
PRESS	642.64	4721.05	8.441E+07
-2 Log Likelihood	63.70	89.17	215.51
BIC	79.09	104.56	230.90
AICc	89.70	115.17	241.51

Table S5 presents the statistical comparison metrics for the model corresponding to each response variable (gel fraction, water absorption rate, and modulus). Model comparison used PRESS (Predicted Residual Sum of Squares), −2 Log Likelihood, BIC (Bayesian Information Criterion), and AICc (corrected Akaike Information Criterion) as key indicators to evaluate the balance between predictive accuracy and model complexity. The PRESS value represents the sum of squared prediction errors; lower values indicate superior predictive performance. PRESS values of 642.6 for gel fraction, 4721.0 for water absorption rate, and 8.44×10^7 for modulus, indicating that residual errors were low across all three responses and that the Quadratic model adequately predicted the experimental data. Notably, the PRESS value for gel fraction was the lowest, suggesting the highest predictive reliability. The −2 Log Likelihood statistic, representing −2 times the log-likelihood of the fitted model, also yielded relatively low values (63.7–215.5) across all responses, indicating good overall model fit. This is consistent with the ANOVA results, which confirm the significance of the Quadratic model ($p < 0.05$). Furthermore, BIC and AICc, which account for model degrees of freedom (df) and parameter numbers, showed lower values (BIC: 79.1–230.9; AICc: 89.7–241.5) than those of the Linear and 2FI models, demonstrating that the Quadratic model achieved an optimal balance between simplicity and explanatory power. Collectively, the comparison of PRESS, BIC, and AICc values indicates that the Quadratic model provided the most appropriate statistical explanatory power and predictive reliability for all responses. This quantitatively supports its designation as the 'Suggested' model in the Fit Summary results (Tables 3–5) of the Design-Expert program.

Table S6. Coded regression coefficients of the quadratic models for gel fraction, water absorption, and modulus

	Gel fraction	Water absorption	Modulus
Intercept	78.26	170.02	5408.22
CMC	8.42	2.87	7866.91
CA	-6.25	-42.14	1330.33
CMC*CA	-0.05	13.70	1783.93
CMC^2	-5.59	-29.08	2780.40
CA^2	0.14	5.90	-304.17

Table S7. Actual regression coefficients of the quadratic models for gel fraction, water absorption, and modulus

	Gel fraction	Water absorption	Modulus
Intercept	55.66	198.35	2545.92
CMC	5.72	16.94	-1023.15
CA	-4.41	-57.63	-577.90
CMC*CA	-0.01	1.83	237.86
CMC^2	-0.22	-1.16	111.22
CA^2	0.06	2.62	-135.19

Equations:

$$\text{Gel fraction} = 55.66 + 5.72 \times CMC + (-4.41) \times CA + (-0.01) \times \mathrm{CMC} \times \mathrm{CA} + (-0.22) \times CMC^2 + 0.06 \times CA^2 \quad \text{(S1)}$$

$$\text{Water absorption} = 198.35 + 16.94 \times CMC + (-57.63) \times CA + 1.83 \times \mathrm{CMC} \times \mathrm{CA} + (-1.16) \times CMC^2 + 2.62 \times CA^2 \quad \text{(S2)}$$

$$\text{Modulus} = 2545.92 + (-1023.15) \times CMC + (-577.90) \times CA + 237.86 \times \mathrm{CMC} \times \mathrm{CA} + 111.22 \times CMC^2 + (-135.19) \times CA^2 \quad \text{(S3)}$$

Table S8. Regression coefficients and confidence intervals of the quadratic model for gel fraction (coded factors)

Factor	Coefficient Estimate	df	Standard Error	95% CI Low	95% CI High	VIF
Intercept	78.26	1	1.71	74.22	82.30	
CMC	8.42	1	1.35	5.22	11.61	1.00
CA	-6.25	1	1.35	-9.44	-3.05	1.00
CMC*CA	-0.0500	1	1.91	-4.57	4.47	1.00
CMC^2	-5.59	1	1.45	-9.01	-2.16	1.02
CA^2	0.1388	1	1.45	-3.29	3.56	1.02

Table S9. Regression coefficients and confidence intervals of the quadratic model for water absorption (coded factors)

Factor	Coefficient Estimate	df	Standard Error	95% CI Low	95% CI High	VIF
Intercept	170.02	1	4.55	159.26	180.78	
CMC	2.87	1	3.60	-5.64	11.37	1.00
CA	-42.14	1	3.60	-50.65	-33.63	1.00
CMC*CA	13.70	1	5.09	1.67	25.73	1.00
CMC^2	-29.08	1	3.86	-38.20	-19.95	1.02
CA^2	5.90	1	3.86	-3.23	15.02	1.02

Table S10. Regression coefficients and confidence intervals of the quadratic model for modulus (coded factors)

Factor	Coefficient Estimate	df	Standard Error	95% CI Low	95% CI High	VIF
Intercept	5408.22	1	586.72	4020.85	6795.59	
CMC	7866.91	1	463.84	6770.09	8963.73	1.00
CA	1330.33	1	463.84	233.51	2427.15	1.00
CMC*CA	1783.93	1	655.97	232.79	3335.06	1.00
CMC^2	2780.40	1	497.42	1604.19	3956.60	1.02
CA^2	-304.17	1	497.42	-1480.37	872.04	1.02

The reliability of the regression coefficients was further verified using 95% confidence intervals (CI) and variance inflation factors (VIF) (Tables S8-10). The VIF values for all factors were close to 1 (1.00–1.02), indicating a very low degree of multicollinearity among the independent variables. Furthermore, the relatively narrow 95% CIs for each coefficient indicate high statistical stability of the estimated parameters. These results support the model's ability to adequately explain the variability observed in the experimental data and to provide reliable predictions. Therefore, the quadratic regression model developed in this study can be regarded as having statistically significant and stable goodness of fit.

Table S11. Confirmation experiment results at the optimal condition (CMC 9 wt% / CA 0.38 wt%)

Analysis	Predicted Mean	Predicted Median	Std Dev	n	SE Pred.	95% PI low	Data Mean	95% PI high
Gel fraction	87.37	87.37	3.82	1	4.87	75.85	87.5	98.88
Water absorption	241.35	241.35	10.18	1	12.97	210.69	210.9	272.02
Modulus	2920.44	2920.44	1311.95	1	1671.77	-1032.67	2967.2	6873.55

Table S12. Confirmation experiment results at the optimal condition (CMC 9 wt% / CA 4 wt%)

Analysis	Predicted Mean	Predicted Median	Std Dev	n	SE Pred.	95% PI low	Data Mean	95% PI high
Gel fraction	72.15	72.15	3.82	1	4.30	61.98	74.7	82.33
Water absorption	133.78	133.78	10.18	1	11.46	106.67	110.0	160.89
Modulus	6434.38	6434.38	1311.95	1	1477.76	2940.03	6137.1	9928.74

Table S13. Point prediction results and confidence intervals for the optimized CMC–CA hydrogel responses (Optimization results within experimental range)

Solution 1 of 100 Response	Predicted Mean	Predicted Median	Std Dev	SE Mean	95% CI low for Mean	95% CI high for Mean	95% TI low for 99% Pop	95% TI high for 99% Pop
Gel fraction	88.7	88.7	3.8	3.9	79.4	98.0	63.5	114.0
Water absorption	256.0	256.0	10.2	10.5	231.2	280.8	188.7	323.3
Modulus	2273.1	2273.1	1312.0	1350.9	-921.2	5467.4	-6404.2	10950.3

Table S14. Point prediction results and confidence intervals for the optimized CMC–CA hydrogel responses (Extended-range optimization results)

Solution 1 of 100 Response	Predicted Mean	Predicted Median	Std Dev	SE Mean	95% CI low for Mean	95% CI high for Mean	95% TI low for 99% Pop	95% TI high for 99% Pop
Gel fraction	89.3	89.3	3.8	4.0	79.9	98.7	63.8	114.8
Water absorption	256.1	256.1	10.2	10.7	230.9	281.4	188.2	324.1
Modulus	2467.1	2467.1	1312.0	1376.8	-788.5	5722.7	-6288.7	11222.9
